# Warming offsets the benefits of elevated CO_2_ in water relations while amplifies elevated CO_2_-induced reduction in forage nutritional value in the C_4_ grass *Megathyrsus maximus*

**DOI:** 10.3389/fpls.2022.1033953

**Published:** 2022-12-05

**Authors:** Eduardo Habermann, Eduardo Augusto Dias de Oliveira, Daniele Ribeiro Contin, João Vitor Campos Pinho Costa, Katia Aparecida de Pinho Costa, Carlos Alberto Martinez

**Affiliations:** ^1^ Department of Biology, Ribeirão Preto School of Philosophy, Science and Literature (FFCLRP), University of Sao Paulo, Ribeirão Preto, SP, Brazil; ^2^ Department of Pharmaceutical Sciences, Ribeirão Preto School of Pharmaceutical Sciences (FCFRP), University of São Paulo, Ribeirão Preto, SP, Brazil; ^3^ Goiano Institute Federal (IF Goiano) at Rio Verde, Rio Verde, GO, Brazil

**Keywords:** climate change, field conditions, grassland, soil moisture conservation, tropical ecosystem

## Abstract

Tropical grasslands are very important to global carbon and water cycles. C_4_ plants have increased heat tolerance and a CO_2_ concentrating mechanism that often reduces responses to elevated concentrations of CO_2_ ([CO_2_]). Despite the importance of tropical grasslands, there is a scarcity of studies that elucidate how managed tropical grasslands will be affected by elevated [CO_2_] and warming. In our study, we used a combination of a temperature-free air-controlled enhancement (T-FACE) and a free-air carbon dioxide enrichment (FACE) systems to increase canopy temperature and [CO_2_] under field conditions, respectively. We warmed a field-grown pasture dominated by the C_4_ tropical forage grass *Megathyrsus maximus* by 2°C above ambient under two levels of [CO_2_] (ambient (*aC*) and elevated (*eC* - 600 ppm) to investigate how these two factors isolated or combined regulate water relations through stomatal regulation, and how this combination affects PSII functioning, biochemistry, forage nutritive value, and digestibility. We demonstrated that the effects of warming negated the effects of *eC* in plant transpiration, water potential, proline content, and soil moisture conservation, resulting in warming canceling the eCO_2_-induced improvement in these parameters. Furthermore, there were additive effects between *eC* and warming for chlorophyll fluorescence parameters and aboveground nutritive value. Warming sharply intensified the eCO_2_-induced decrease in crude protein content and increases in forage fibrous fraction and lignin, resulting in a smaller forage digestibility under a warmer CO_2_-enriched atmosphere. Our results highlight the importance of multifactorial studies when investigating global change impacts on managed ecosystems and the potential consequences for the global carbon cycle like amplification in methane emissions by ruminants and feeding a positive climate feedback system.

## Introduction

Atmospheric CO_2_ concentration ([CO_2_]) and temperature are critical components of global carbon and water cycles. Anthropogenic greenhouse emissions are expected to increase atmospheric [CO_2_] to 600 ppm by 2050 with a simultaneous warming of the troposphere of +2°C above pre-industrial values (IPCC, 2021). This unequivocal influence of anthropic activity on climate is already causing significative disturbances on natural (Brienen et al., 2015) and managed ecosystems (Jägermeyr et al., 2021; Spera et al., 2020). The impacts of human-induced climate change will presumably be more intense in tropical and subtropical developing countries (Chapagain et al., 2020) where agriculture and livestock are the main economic activities. In tropical and subtropical countries, meat and milk production greatly depends on productivity and nutritive value of rainfed grasslands that will likely be affected by climate change (Habermann et al., 2021). Therefore, understanding how the combination of increased levels of [CO_2_] and temperature will impact tropical ecosystems is paramount.

In general, elevated [CO_2_] (*eC*) and elevated temperature (*eT*) have contrasting effects on plant transpiration and water relations (Ainsworth and Long, 2021). On the one hand, *eC* often reduces stomatal conductance (*g_s_*) and transpiration rates (*E*) by a combination of short-term mechanisms (stomatal closure) and long-term changes (decreased stomatal density) (Xu et al., 2016). As a consequence, evapotranspiration is reduced improving plant water status and conserving soil moisture (Leakey et al., 2006; Markelz et al., 2011). Moreover, *eC* improves PSII performance, and photosynthetic rates (Habermann et al., 2019a), and alters the metabolism of proline (Shabbaj et al., 2022) and antioxidant enzymes (Pritchard et al., 2000). On the other hand, *eT* increases the permeability of K^+^ to guard cells and promotes H^+^ efflux from guard cells, leading to water influx and stomata opening (Ilan et al., 1995). As a consequence, evapotranspiration increases reducing soil moisture (Xu et al., 2013). The final effects of the combination of *eC* and *eT* will depend on a balance of the antagonistic forces over plants at the canopy and leaf level and may be affected by other biotic and abiotic factors. Despite the importance of multifactorial studies to investigate the combined effects of *eC* and *eT* on the performance of pastures, there is a paucity of leaf or canopy level studies in the literature, limiting our understanding of global change effects on pastures.

In the context of livestock, any impact of global changes on the chemical composition of leaves may change the potential acquisition of energy by animals, possibly impacting the global food production chain and food security (Craine et al., 2010). Ruminants obtain energy from plant material especially from sugars, proteins and other easily digestible compounds, while leaf fibrous fraction and lignin are anti-quality components that prevent forage from being degraded, decreasing the digestibility of food (Waghorn and Clark, 2004). Some studies (Habermann et al., 2019a) and meta-analyses (Lee et al., 2017; Moyo and Nsahlai, 2021) have indicated that future atmospheric conditions will decrease forage nutritive value by elevating lignin and fibrous fraction levels, while decreasing forage crude protein content, even under well-watered conditions (Habermann et al., 2019a). Moreover, elevated [CO_2_] is also expected to decrease forage nutritive value by decreasing protein levels and increasing fibrous fraction and lignin (Augustine et al., 2018). Therefore, future *eC* and *eT* conditions may synergize to produce pastures with reduced nutritive value and digestibility, impacting cattle weight gain, milk production, and the methane emissions by ruminants.

Evaluating plant performance under predicted global changing conditions has some methodological challenges that arrive from physical barriers used to control atmospheric conditions (Miglietta et al., 1999; Kimball, 2005). Some of these challenges are related to the artificial atmospheric conditions inside growth chambers or open top chambers such as modified radiation intensity and wind speed, and magnified vapor pressure deficit (Leadley et al., 1997; Uprety et al., 2006), whilst other challenges are related to limited plant growth, such as limited root development in pots or plant size in growth chambers. All these limitations may lead to different plant behaviors between controlled and field conditions (Macháčová, 2010). Here, we used a combination of a Temperature Free-Air Controlled Enhancement system (T-FACE) and a mini free-air CO_2_ enrichment system (mini-FACE) to warm the canopy and to increase atmospheric [CO_2_], respectively, in open field conditions. Until this moment, the majority of FACE and T-FACE field facilities are located in temperate regions, creating a knowledge gap in tropical and subtropical regions.

*Megathyrsus maximus* cv. Mombaça (C_4_) (popularly known as Guinea grass) is a perennial bunchgrass native to Africa and widely cultivated in Brazil for animal feeding (Hitchcock and Chase, 1910), covering around 8 million hectares of Brazilian territory. This cultivar is adapted to fertile soils (Embrapa, 2014) and has total dry matter productivity of 33 t/ha/year (Embrapa, 2012). Here, we warmed a field-grown pasture of *M. maximus* cv. Mombaça under ambient and elevated [CO_2_] using a T-FACE and FACE system to investigate how elevated [CO_2_] and warming interact to regulate water relations of a managed field-grown pasture of *M. maximus*, and how this interaction will affect PSII functioning, biochemistry, forage nutritive value, and digestibility. We hypothesized that warming will counteract the beneficial effects of elevated [CO_2_] on plant water relations while amplifying the negative effects of *eC* on forage nutritive value.

## Material and methods

### Experimental site

The experiment was conducted under field conditions at Trop-T-FACE facility located at the University of São Paulo at Ribeirão Preto, São Paulo State, Brazil (21° 9’42.56” S, 47° 50’ 47.12” W) from September/2015 to December/2015. The climate in this region is classified as B2rB’4a’ (moist meso-thermal with small water deficiency) (Thornthwaite, 1948) and the soil is Rhodic Ferralsol with clay texture (IUSS, 2015). Two months before planting, we conducted a soil chemical analysis using soil samples (20 cm depth) from all 16 plots to correct any nutritional deficiency or pH differences between plots. To raise soil pH to 5.5 we used calcined limestone (48% CaO and 16% MgO) and to correct soil nutritional deficiency between plots we conducted a differential soil fertilization for each plot according to Raij et al. (1997).

Seeds of *Megathyrsus maximus* (Syn. *Panicum maximum*) cv. Mombaça were sowed on 10 × 10 m plots and seedlings were irrigated until pasture establishment by a sprinkler irrigation system. Irrigation automatically watered plants in the early morning (6 am) to maintain soil water content (*SWC*) around 0.45 m^3^ m^-3^. After pasture establishment (which occurred approximately 2 months after sowing), plants reached 90 cm height and were clipped at 30 cm above the ground to simulate grazing. After clipping, treatments were immediately applied during 30 days of vegetative regrowth. In Brazil, rainfed pastures represent the most common feed source for ruminants. Therefore, during the experimental period, no additional irrigation was applied and plants were rainfed. We used an automatic climate station WS-PH1 connected to a data logger DL2e (Delta-T Devices, Cambridge, UK) to monitor and store the meteorological data. We monitored the *SWC* and soil temperature (*Tsoil*) hourly during the entire experiment using theta Probe ML2X and ST2 sensors, respectively, connected to a DL2e datalogger (Delta-T Devices Ltd., Burwell, Cambridge, UK) located in the center of each plot at 10-cm depth. During the growing season, a high frequency of rainfall was observed, totalizing 224 mm of total precipitation. The average total solar radiation was 0.33 kW m^-2^ with maximum values of 1.1 kW m^-2^ at the midday period. The average relative air humidity was 87% with minimum values of 38%. Average air temperature, maximum air temperature, and minimum air temperature were 25°C, 35°C, and 16°C, respectively.

In this experiment, we tested the effects of two levels of canopy temperature: ambient (*aT*) and elevated (*eT*, +2°C above ambient) under two atmospheric CO_2_ concentration levels: ambient CO_2_ concentration (*aC*, plots with ambient [CO_2_]) and elevated CO_2_ concentration (*eC*, 200 ppm above ambient CO_2_ concentration). We combined both factors with two levels each in four treatments (*aCaT*, *eCaT*, *aCeT*, and *eCeT*) in a randomized blocks design. The experiment was conducted with four replicates (16 plots, n = 4 for each treatment).

### Elevated temperature treatment

The plant canopy was warmed by a T-FACE system (temperature-free air-controlled enhancement system) 24 hours per day during the entire experiment as described by Kimball (2005). In each warmed plot, six 750 W infrared heaters, model FTE-750-240 Salamander ceramic infrared heating element (Mor Electric Heating, Comstock Park, MI, USA) located at 0.8 m above the canopy in a hexagonal arrangement were responsible for warming the plants. We installed each heater on an aluminum reflector Salamander ALEX (Mor Electric Heating, Comstock Park, MI, USA). We also installed six reflectors in each non-warmed plot with no infrared heater (dummy heaters) to simulate the shading (nearly negligible) between warmed and non-warmed plots (around 1% of shading). To control the canopy temperature between plots, T-FACE system used a proportional-integrative-derivative (PID) control system installed in a datalogger model CR1000 with AM2 5 T multi- plexors (Campbell Scientific, Logan, UT, USA). Canopy temperature was constantly monitored by infrared radiometers model SI-1H1-L20 (Apogee Instruments, Logan, UT, USA) located in each plot. In this study, we used the 2°C above ambient canopy temperature set-point during the entire experiment and 24 h per day.

### Elevated CO_2_ treatment

We used a mini free-air CO_2_ enrichment system (mini-FACE) (Miglietta et al., 1999) to increase the atmospheric [CO_2_] concentration by 200 ppm above ambient [CO_2_] under field conditions. In each *eC* plot, one PVC ring of 2 m diameter punctured with micro holes fumigated pure CO_2_ within the plant canopy. ‘Dummy’ rings were installed in each *aC* plot. Each ring was placed within the 10 × 10 m plots to minimize the edge effect. To monitor the atmospheric [CO_2_] concentration in each plot, we installed one GMT222 CO_2_ transmitter sensor (Vaisala, Helsinki, Finland) at canopy height in the central region of each plot. Using a proportional integration device algorithm (PID) and [CO_2_] data of *aC* and *eC* plots, FACE central unit regulated the amount of [CO_2_] needed in *eC* plots to increase atmospheric [CO_2_] by 200 ppm above ambient [CO_2_]. Wind speed was measured by an anemometer located in the central region of the experimental site 3 m above the ground. The liquid pure CO_2_ fumigated in each plot was stored within a 12-ton cryogenic tank with a vaporizer unit. The 200 ppm above ambient [CO_2_] target was maintained from sunrise to sunset.

### Leaf gas exchange and water relations

We measured leaf gas exchange in the central region of 4 fully expanded leaves per plot, between 9 and 11 am, 22 days after the treatments started (DATS). Measurements were conducted with a LCProSD^+^ advanced photosynthesis measurement system (ADC BioScientific, UK). Leaves were kept in the chamber until the variables remained stable. During measurements we used constant conditions of radiation (1740 μmol m^-2^ s^-1^), temperature of 30°C (*aT* plots) or 32°C (*eT* plots), and [CO_2_] of 400 μmol mol^-1^ (*aC* plots) or 600 μmol mol^-1^ (*eC* plots). We measured the net photosynthesis rate (*A*), stomatal conductance (*g_s_*), and transpiration rate (*E*).

Xylem water potential (Ψ_w_) was measured using a Scholander chamber model 3005 (Soil moisture Equipment Corp., Santa Barbara, CA, USA) (Scholander et al., 1965) at 16, 23, and 25 DATS at the midday period. At 27 DATS, a diurnal course of Ψ_w_ was conducted every 4 hours from 5 am to 5 pm. For Ψ_w_ measurements, 3 fully expanded leaves were detached from each plot from different plants and placed within sealed plastic bags containing a wet filter paper. Then, samples were taken to Scholander chamber where measurements were performed (this process took approximately 3 minutes). Leaf relative water content (*RWC*) was measured at 16, 23, and 25 DATS at the midday period using leaf discs as described by Smart and Bingham (1974). For *RWC* measurements, 3 fully expanded leaves were detached from each plot from different plants and placed within sealed plastic bags containing a wet filter paper. Ten leaf discs were detached from the central region of each leaf and immediately weight to measure the fresh weight (FW). Then, samples were submerged in distilled water for 24 h at 4°C in the dark. Samples were dried to remove water from the leaf surfaces and weight to obtain the turgid weight (TW). Finally, all plant material was dried at 60°C until constant dry weight in a fan-forced oven to determine the dry weight (DW). *RWC* was calculated according to the equation: *RWC* = FW-DW/TW- DW × 100.

### Leaf proline content

Three fully expanded leaves were detached from each plot from different plants and immediately placed on liquid nitrogen. Samples were kept at -20°C until analysis. Leaf proline content (Pro) was measured according to Bates et al. (1973) at 16, 23, and 25 DATS at the midday period. At 27 DATS, a diurnal course of Pro was conducted every 4 hours from 5 am to 5 pm. In summary, 200 mg of fresh leaves were macerated in liquid nitrogen and homogenized in 10 mL of sulphosalicylic acid (3%). This solution was filtered and 2 mL of filtered solution was placed in glass tubes. In each tube, we added 2 mL of acid ninhydrin (1.25 g of ninhydrin, 30 mL of glacial acetic acid, and 20 mL of phosphoric acid 6M). The tubes were maintained in a stove during 1 hour at 100°C and immediately put on ice to stop the reaction. Then, 4 mL of toluene was added to each tube and the solution was mixed in a vortex for 20 seconds. From the two-phase system formed in each tube, the upper layer (chromophore) was used for the measurements at 520 nm.

### Antioxidant enzymes and lipid peroxidation

We collected 3 fully expanded leaves per plot at midday period from different plants at the end of the experimental period to measure the activity of antioxidant enzymes and the level of lipid peroxidation. Leaves were immediately placed in liquid nitrogen and maintained at -20°C until analysis. We used the method described by Azevedo et al. (1998) to obtain the crude extracts. In summary, 1 g of fresh leaves was macerated with liquid nitrogen and homogenized with 10 mL of potassium phosphate buffer (100 mM, pH 9.0) containing EDTA (1 mM), DTT (3 mM) and PVPP (2%, m/v). Crude protein content was measured according to Bradford (1976) at 595 nm. We measured the activity of four enzymes: catalase (CAT, EC 1.11.1.6) using the spectrophotometric method described by Azevedo et al. (1998), in which the results were expressed as μmol min^-1^ mg^-1^ protein using the molar extinction coefficient 40.0 M^-1^ cm^-1^. Ascorbate peroxidase (APX, EC 1.11.1.11) was determined using the Nakano and Asada (1981) method. APX activity was expressed in μmol min^-1^ mg^-1^ protein. We used the molar extinction coefficient 3051.4 M^-1^ cm^-1^, determined for our experimental conditions. Superoxide dismutase (SOD, EC 1.15.1.1) was measured as suggested in Giannopolitis and Ries (1977). In this study, a SOD unit was defined as the amount of enzyme that inhibits NBT photoreduction by 50% (Beauchamp and Fridovich, 1971). The activity of non-specific peroxidases (POD, EC 1.11.1.7) according to Zeraik et al. (2008) using guaiacol as substrate. The results were expressed in μmol min^-1^ mg^-1^ of protein, using the coefficient of molar extinction 26.6 mM^-1^ cm^-1^ (Chance and Maehly, 1955) in our calculations. We measured the level of lipid peroxidation of lead tissues determining the level of malondialdehyde (MDA) according to the thiobarbituric acid (TBA) method as described by Heath and Packer (1968).

### Photosynthetic pigments

On the last day of the experimental period, we collected 3 fully expanded leaves per plot at the midday period (between 12 am and 1 pm) and immediately put the samples on liquid nitrogen. Samples were maintained at -20°C until analysis. We used a method adapted from Lichtenthaler and Wellburn (1983). Here, 100 mg of fresh leaves were macerated in 5 mL of 80% acetone solution and centrifuged to precipitate the biggest particles. Supernatant absorbance was measured with a spectrophotometer at 480, 645 and 663 nm. We determined chlorophyll a, chlorophyll b, and carotenoid content and calculated the total chlorophyll content (a + b) and chlorophyll/carotenoid.

### Chlorophyll fluorescence

We measured chlorophyll fluorescence parameters from 3 fully expanded leaves per plot between 9 am and 11 am on the last day of the experiment using an imaging-PAM M-series chlorophyll fluorescence system (MINI-version model, Heinz Walz GmbH, Germany). Leaves were detached, maintained in water and dark-acclimated for 30 minutes. We measured dark fluorescence yield (Fo) using a low frequency of pulse-modulated measuring light (0.5 μmol m^−2^ s^−1^, 100 μs, 1 Hz) and maximum fluorescence yield (Fm) using a saturation pulse (2700 μmol m^−2^ s^−1^, 0.8 s, 10 Hz). Then, leaves were submitted to increasing steps of photosynthetic photon flux density (PPFD) levels (from 0 to 784 μmol photons m^−2^ s^−1^). We measured the effective PS II quantum yield during illumination Y(II), the quantum yield of non-regulated energy dissipation Y(NO), the quantum yield of regulated energy dissipation Y(NPQ) and maximum electron transport rate (ETR_max_). Parameters were calculated as follows: Y(II) = (Fm’ − F)/Fm’, Y(NO) = 1/(NPQ + 1 + qL(Fm/Fo − 1), Y(NPQ) = 1 - Y(II) - 1/(NPQ+1+qL(Fm/Fo-1)), NPQ = (Fm − Fm’)/Fm’ and ETR = 0.5 × PSII yield × PAR × 0.84 equivalents m^−2^ s^−1^. Results were analyzed at 784 μmol photons m^−2^ s^−1^.

### Forage quality and digestibility

At the end of the experimental period, we harvested the aboveground material 30 cm aboveground. Plant material was oven dried at 70°C until constant weight, grounded in an electric mill (Willey type), and used to measure forage nutritive value parameters. Neutral detergent fiber (NDF) and acid detergent fiber (ADF) were determined according to the method described by Mertens (2002) using fiber determination equipment (TE-149, Tecnal). We measured nitrogen content (N) using a nitrogen distiller (TE-0363, Tecnal) and calculated the crude protein content (CP) using the equation CP = N × 6.25 (AOAC, 1990). Using the forage ADF fraction, samples were submitted to acid hydrolysis with sulfuric acid 72% as described in Silva and Queiroz (2002) to determine lignin content. In-vitro dry matter digestibility (IVDMD) was measured according to Tilley and Terry (1963) that was adjusted to artificial rumen by ANKON^®^ using the “Daisy incubator” device from Ankon Technology (*in vitro* true digestibility).

### Statistical analysis

Data were checked for outliers and normality. We used a two-way ANOVA with two factors (with two levels each) to evaluate the isolated effects of [CO_2_] and temperature and the interaction between factors. Comparisons between two average values were conducted using a student t-test. Regressions analysis was used to investigate the relations between parameters. Statistical analysis was conducted using Past and GraphPad Prism 9 software.

## Results

### Microclimate

**Figure 1:**
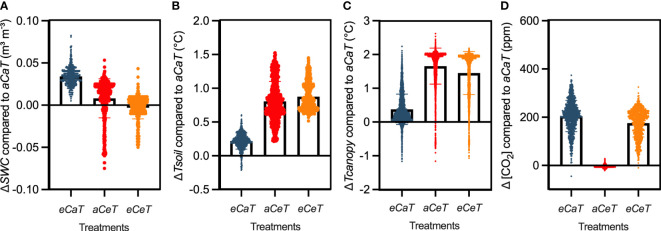
Difference (Δ) in the microclimate of each treatment compared to ambient conditions (*aCaT*). **(A)** *SWC* = soil water content (m^3^ m^-3^), **(B)** *Tsoil* = soil temperature (°C). **(C)** *Tcanopy* = canopy temperature (°C). **(D)** [CO_2_] = atmospheric CO_2_ concentration. Bars show the average difference between treatments and *aCaT*, whilst each point shows the dispersion around the average value. Points were obtained each hour for *SWC* and *Tsoil* and every 15 minutes for *Tcanopy* throughout the entire experiment. For [CO_2_], each point represents a 5-minute average value along six days of the experimental period during the diurnal period (6 am to 6 pm - fumigation period). Treatments: *aC* (ambient CO_2_ concentration), *eC* (elevated CO_2_ concentration – 600 ppm), *aT* (ambient temperature), and *eT* (elevated temperature - 2°C above ambient temperature).

The effects of treatments on the microclimate conditions are shown in **Figure 1** and more details can be found on **Supplementary Figure 1**. We observed that *SWC* under *eCaT* was higher than under *aCaT* during the entire experimental period, resulting in the distribution of *SWC* values observed in **Figure 1A**. However, this water conservation effect was completely offset by warming (**Figure 1A**). *Tsoil* was higher under *eT* plots regardless [CO_2_] level (**Figure 1B**). On average, *Tcanopy* at *eCaT* treatment was warmer than under *aCaT*, especially during the warmest period of the day. In warmed plots, T-FACE system maintained *Tcanopy* 2°C above ambient, especially during the night period (**Supplementary Figure 1**). However, during the day, variations among the set-point were observed, leading to an average warming of approximately 1.5°C above ambient (**Figure 1C**). This behavior is expected in open-field warming systems due to the higher evapotranspiration and wind speed, especially during the mid-day period (Kimball, 2005). Average [CO_2_] on *aCaT*, *eCaT*, *aCeT*, and *eCeT* plots were 406 ± 45, 402 ± 46, 611 ± 49, and 581 ± 43 ppm, respectively (**Figure 1D**). [CO_2_] was 404 and 596 ppm under *aC* and *eC* plots, respectively (**Figure 1D**).

**Figure 2:**
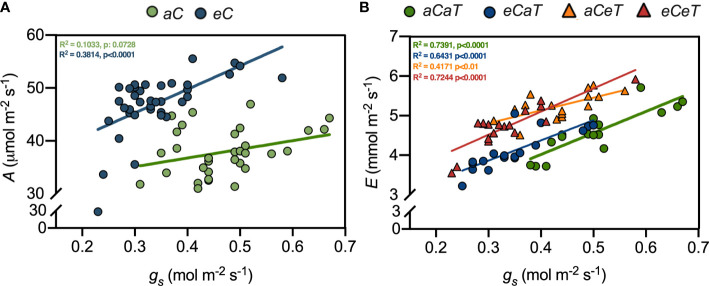
Linear regressions between leaf gas exchange parameters measured in fully expanded leaves of *M. maximus* at different levels of [CO_2_] and temperature. *A* = net photosynthesis rate. *g_s_* = stomatal conductance. *E* = transpiration rate. R^2^ and p-values are shown for each regression line according to the color of each treatment. **(A)** Equations: *aC* = Y = 16,40*X + 30,19, *eC* = Y = 44,53*X + 31,92. **(B)** Equations: *aCaT* = Y = 5,437*X + 1,843, *eCaT* = Y = 5,100*X + 2,334, *aCeT* = Y = 3,237*X + 3,837, and *eCeT* = Y = 5,845*X + 2,763. Regressions were performed using the raw data from Habermann et al. (2019). Treatments: *aC* (ambient CO_2_ concentration), *eC* (elevated CO_2_ concentration – 600 ppm), *aT* (ambient temperature), and *eT* (elevated temperature - 2°C above ambient temperature).

### Gas exchange and water relations

**Figure 3:**
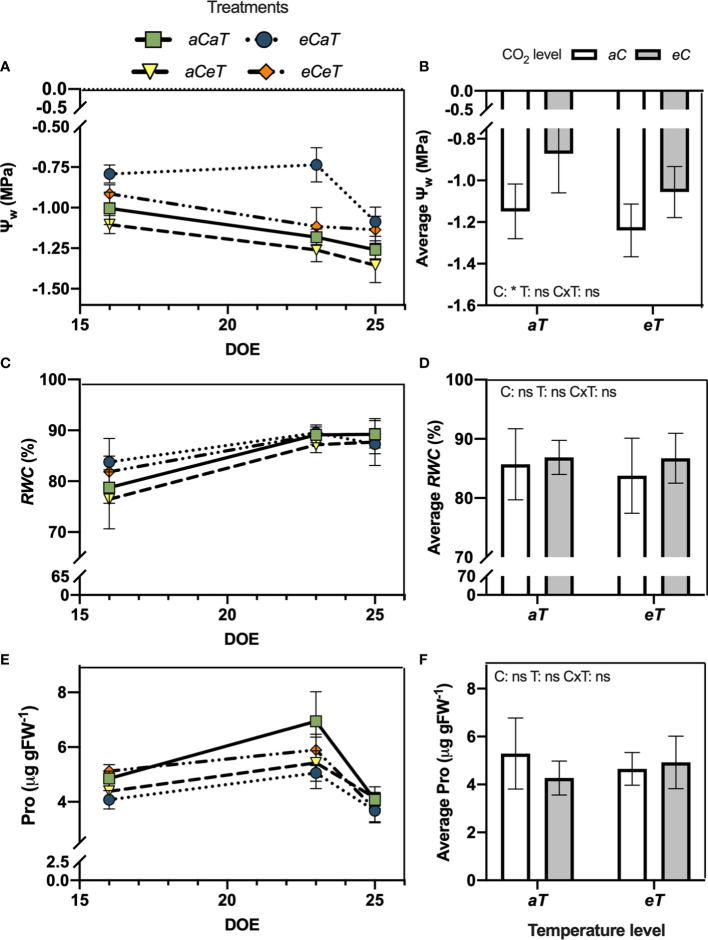
Water status and leaf proline content of fully expanded leaves of *M. maximus* during the experimental period in different levels of [CO_2_] and temperature (left column) and average values (right column). Ψ_w_ = water potential. *RWC* = relative water content. Pro = leaf proline content. DOE = day of the experiment. Treatments: *aC* (ambient CO_2_ concentration), *eC* (elevated CO_2_ concentration – 600 ppm), *aT* (ambient temperature), and *eT* (elevated temperature - 2°C above ambient temperature). ANOVA results are shown for diurnal averages: C: [CO_2_] effect, T: temperature effect, C×T: interaction between C and T. *P < 0.05, **P < 0.01, ***P < 0.001, ns, non-significant.

In general, *eC* improved photosynthesis regardless of temperature level (**Figure 1**, **Supplementary Table 1**), while *eC* and *eT* showed antagonistic effects on plant transpiration and water relations (**Figures 1**-**3**). Since ANOVA analysis revealed no effects of temperature on photosynthesis and stomatal aperture, we investigated the association between *A* and *g_s_* under both levels of [CO_2_]. As expected, higher values of *A* associated with smaller *g_s_* were observed under *eC* (**Figure 1A**). However, we observed antagonistic effects between *eC* and *eT* for *E*. Therefore, associations between *g_s_* and *E* were made for each treatment and *eT* completely mitigated the CO_2_-induced reduction of *E* (**Figure 1B**).

At 16 DATS, Ψ_w_ increased (smaller negative values) under *eC* regardless of temperature level by approximately 21% (**Figure 3A**). At 23 DATS, we observed antagonistic effects between CO_2_ and warming, leading to a Ψ_w_ similar to *aCaT* treatment. At 25 DATS, *eC* regardless of temperature level increased Ψ_w_ by 13% (**Figure 3A**). Therefore, the diurnal average Ψ_w_ was higher under *eC* regardless of temperature level (**Figure 3B**). However, no differences in *RWC* were observed during the experiment (**Figure 3C**) or in the diurnal average value (**Figure 3D**) as a result of CO_2_ enrichment or warming. PRO content was also not modified during the experiment (**Figure 3E**) or in the diurnal average value (**Figure 3F**).

**Figure 4:**
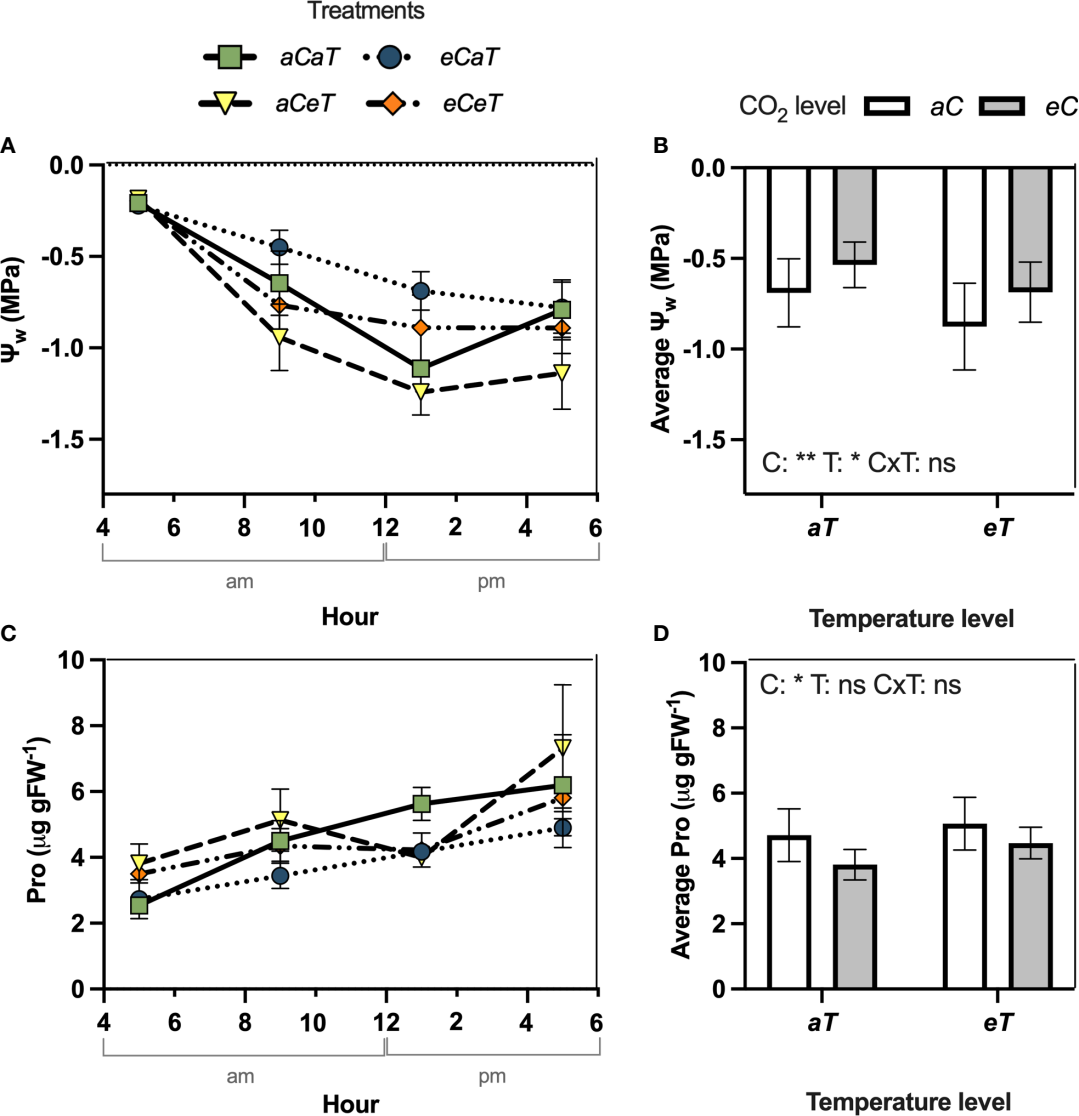
Diurnal course of water potential and leaf proline content measured in fully expanded leaves of *M. maximus* in different levels of [CO_2_] and temperature during the diurnal period (left column) and diurnal average values (right column). Ψ_w_ = water potential. Pro = leaf proline content. Treatments: *aC* (ambient CO_2_ concentration), *eC* (elevated CO_2_ concentration – 600 ppm), *aT* (ambient temperature), and *eT* (elevated temperature - 2°C above ambient temperature). ANOVA results are shown for diurnal averages: C: [CO_2_] effect, T: temperature effect, C×T: interaction between C and T. *P < 0.05, **P < 0.01, ***P < 0.001, ns, non-significant.

Additional measurements conducted during an entire diurnal course revealed that the pattern of response observed during the experimental period was similar to those observed in the diurnal course (**Figure 4**). In the diurnal course conducted at 27 DATS, we observed that Ψ_w_ increased by 38% (p=0.015) at 1 pm under *eC* regardless of the temperature level (**Figure 4A**). During the entire diurnal course, we observed that Ψ_w_ remained higher (smaller negative values) under *eC*, resulting in a daily average Ψ_w_ 23% higher (P<0.05) than under *aC* regardless of the temperature level (**Figure 4B**). However, at 9 am and 5 pm Ψ_w_ decreased (P<0.05) due to isolated effects of *eT* in 45% and 44%, respectively, regardless CO_2_ level (**Figure 4A**), resulting in a daily average Ψ_w_ 25% smaller (higher negative values) (**Figure 4B**). Under *eCeT*, the antagonistic effects of CO_2_ and warming resulted in a diurnal average Ψ_w_ not different when compared to *aCaT* conditions (**Figure 4B**). During the diurnal course, Pro increased under *eT* regardless CO_2_ level by approximately 50% at 5 am (P<0.05) (**Figure 4C**), but with no significant results in Pro diurnal average (**Figure 4D**). On the other hand, *eC* regardless of temperature level decreased (P<0.05) Pro diurnal average by 19% (**Figure 4D**).

### Chlorophyll fluorescence and biochemical parameters

We observed that ETR increased by additive effects of *eC* and *eT* resulting in an ETR approximately 60% higher under *eCeT* when compared to *aCaT* (**Table 1**). Additive effects between *eC* and *eT* were also observed for Y(II). In addition, qP and qL both increased under *eC* regardless of temperature level. Y(NPQ) and Y(NO) were not affected by [CO_2_] or temperature levels. CAT, POD, and SOD activities and MDA content were also not changed as a result of treatments (**Table 1**). However, APX activity decreased under *eT* regardless [CO_2_] level. A few interactions between *eC* and *eT* were observed for photosynthetic pigments, but no significant difference was observed between *eCeT* and *aCaT* average values (**Table 1**).

**Table 1:** Chlorophyll fluorescence parameters, enzymatic activity, malondialdehyde levels, and photosynthetic pigments content measured on the last day of the experimental period in fully expanded leaves of *M. maximus* developed at two levels of [CO_2_] and temperature.

Parameter	Treatments				ANOVA		
	*aCaT*	*eCaT*	*aCeT*	*eCeT*	C	T	CxT
ETR	36.3 ± 3.3	51.5 ± 3.4	47.7 ± 4.9	58.3 ± 4.6	*	*	ns
Y(NPQ)	0.95 ± 0.05	0.87 ± 0.05	0.85 ± 0.11	0.75 ± 0.02	ns	ns	ns
qL	0.21 ± 0.03	0.26 ± 0.02	0.20 ± 0.03	0.29 ± 0.009	**	ns	ns
qP	0.29 ± 0.03	0.36 ± 0.02	0.30 ± 0.03	0.41 ± 0.011	**	ns	ns
Y(II)	0.10 ± 0.009	0.14 ± 0.010	0.13 ± 0.013	0.17 ± 0.006	**	*	ns
Y(NO)	0.19 ± 0.004	0.19 ± 0.009	0.21 ± 0.017	0.21 ± 0.004	ns	ns	ns
CAT	30.1 ± 4.9	31.5 ± 1.6	29.8 ± 2.7	29.1 ± 4.4	ns	ns	ns
APX	0.32 ± 0.03	0.36 ± 0.04	0.26 ± 0.03	0.29 ± 0.05	ns	*	ns
POD	1.7 ± 0.17	2 ± 0.22	1.4 ± 0.04	1.7 ± 0.17	ns	ns	ns
SOD	30.8 ± 0.9	29.4 ± 3.9	25.7 ± 2.1	27.6 ± 3.5	ns	ns	ns
MDA	259.4 ± 32.8	283.8 ± 21.6	295.5 ± 17.2	283.2 ± 14.6	ns	ns	ns
Chl a	5.3 ± 0.4	4.5 ± 0.3	4.8 ± 0.2	5.2 ± 0.3	ns	ns	*
Chl b	1.9 ± 0.1	1.6 ± 0.1	1.7 ± 0.1	1.8 ± 0.1	ns	ns	*
Carot	2.8 ± 0.2	2.5 ± 0.1	2.6 ± 0.1	2.6 ± 0.1	ns	ns	ns
Chl a+b	7.2 ± 0.5	6.2 ± 0.4	6.6 ± 0.3	7.3 ± 0.2	ns	ns	*
Chl a/b	2.8 ± 0.07	2.7 ± 0.05	2.8 ± 0.03	2.7 ± 0.05	ns	ns	ns
Chl/Carot	2.6 ± 0.03	2.5 ± 0.01	2.6 ± 0.04	2.5 ± 0.02	ns	ns	ns

ETR , electron transport rate; Y(NPQ) , quantum yield of regulated energy dissipation; qL , photochemical quenching (lake model); qP , photochemical quenching (puddle mode); Y(II) , effective PS II quantum yield during illumination; Y(NO) , quantum yield of non-regulated energy dissipation; CAT , catalase activity; APX , ascorbate peroxidase activity; POD , non-specific superoxide activity; SOD , superoxide dismutase activity; MDA , leaf malondialdehyde level; Chl a , Chlorophyll a concentration; Chl b , Chlorophyll b concentration; Carot , carotenoid concentration. ANOVA results are shown for each parameter, C, [CO_2_] effect; T, temperature effect; C×T, interaction between C and T. *P < 0.05, **P < 0.01, ***P < 0.001, ns, non-significant. Fluorescence parameters were analyzed at 784 μmol photons m^−2^ s^−1^.

### Forage nutritive value and digestibility

**Figure 5:**
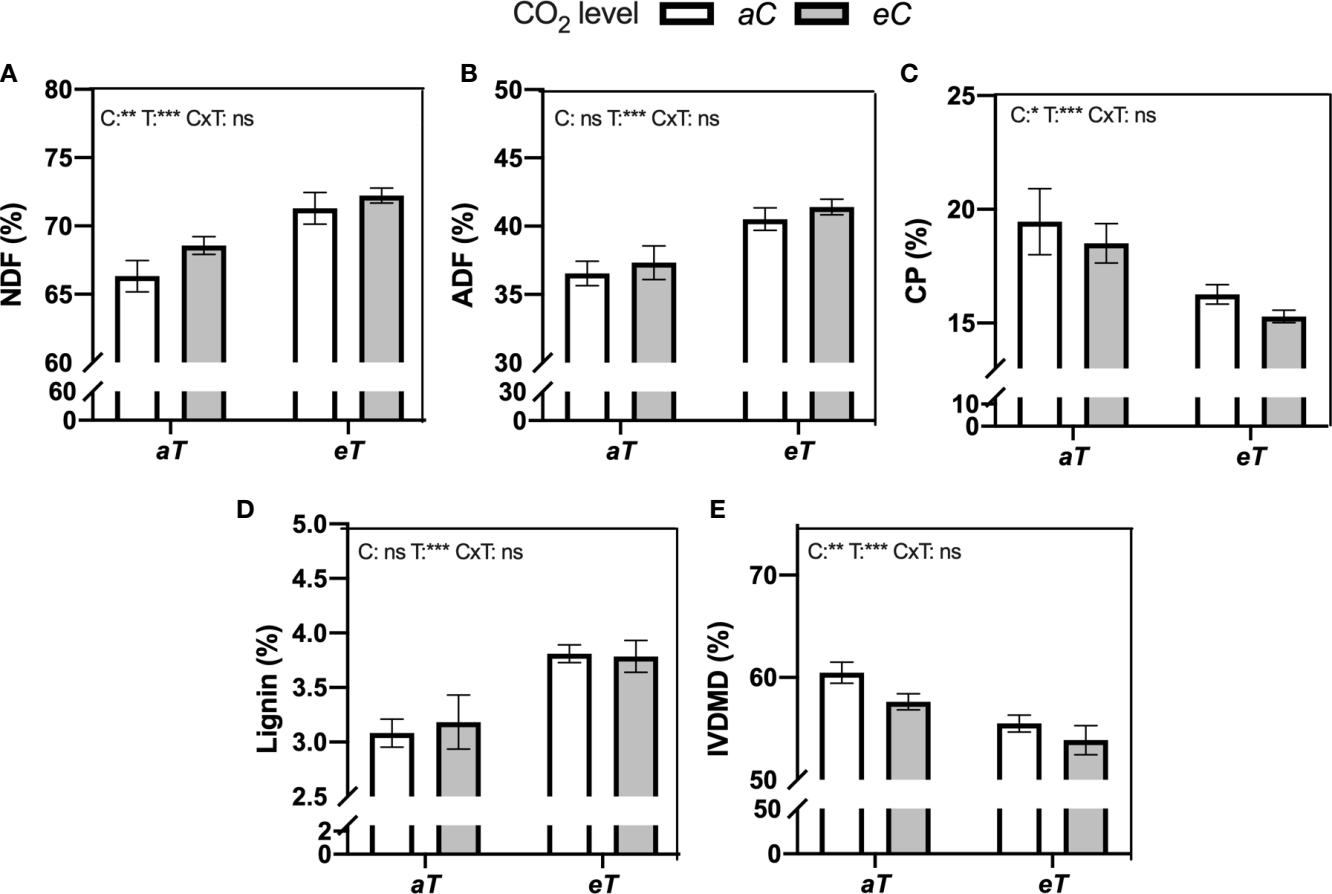
Forage nutritional value of the aboveground dry mass of *M. maximus* measured in different levels of [CO_2_] and temperature. NDF , neutral detergent fiber. ADF , acid detergent fiber. CP , crude protein. IVDMD , in-vitro dry matter digestibility. Treatments: *aC* (ambient CO_2_ concentration), *eC* (elevated CO_2_ concentration – 600 ppm), *aT* (ambient temperature), and *eT* (elevated temperature - 2°C above ambient temperature). ANOVA results are shown for each parameter: C, [CO_2_] effect; T, temperature effect; C×T, interaction between C and T. *P < 0.05, **P < 0.01, ***P < 0.001, ns, non-significant.

Significative additive effects between *eC* and *eT* were observed for forage nutritive value parameters (**Figure 5**). Forage fiber fraction increased due to *eC* and *eT* effects, resulting in NDF and ADF values 14% and 13.4% higher under *eCeT* when compared to *aCaT*, respectively (*student t-test comparison*) (**Figures 5A, B**). CP decreased more under combined conditions (*eCeT*), than under isolated effects, resulting in 21.2% less CP available in the forage material under *eCeT* compared to *aCaT* (**Figure 5C**). Lignin content increased by warming regardless [CO_2_] level by approximately 21% when compared to *aT* conditions (**Figure 5D**). As result of higher forage fiber fraction, lignin, and less CP, IVDMD decreased by 4.7%, 8.2%, and 11% under *eCaT*, *aCeT*, and *eCeT*, respectively, when compared to *aCaT* (*additive effect*) (**Figure 5E**).

**Figure 6:**
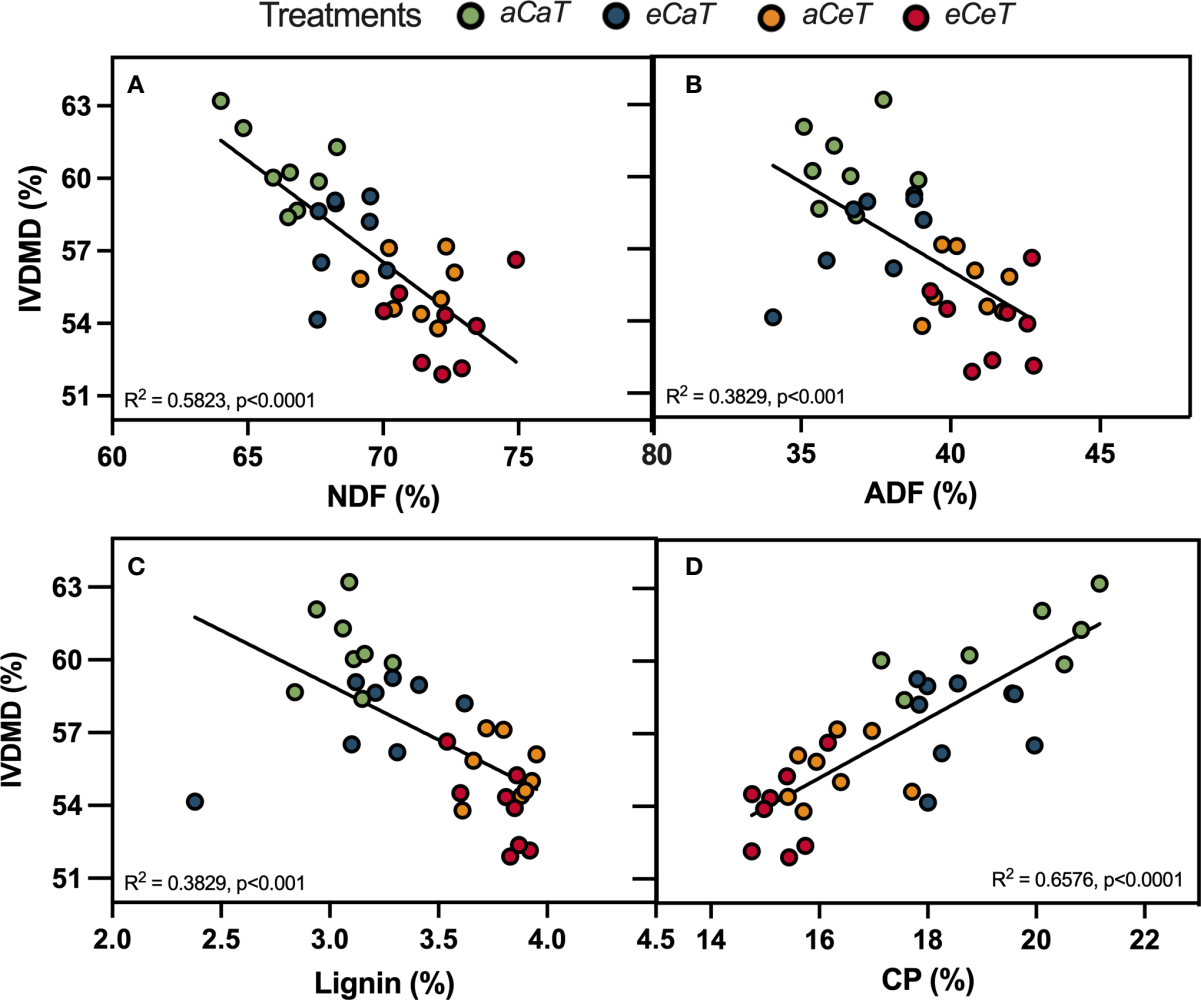
Linear regressions between for age nutritive value parameters and in-vitro dry matter digestibility (IVDMD) of *M. maximus* above ground dry mass measured at different levels of [CO2] and temperature. **(A)** NDF, neutral detergent fiber. **(B)** ADF, acid detergent fiber. **(C)** Lignin. **(D)** CP, crude protein. R2 and p-values are shown for each regression line. Treatments: *aC* (ambient CO_2_ concentration), *eC* (elevated CO_2_ concentration – 600 ppm), *aT* (ambient temperature), and *aT* (elevated temperature - 2°C above ambient temperature).

We observed linear relations between forage nutritive value parameters and IVDMD (**Figure 6**). IVDMD decreased as ADF, NDF, and lignin content increased (**Figures 6A–C**), whilst a higher IVDMD was observed as CP increased (**Figure 6D**).

## Discussion

It is widely reported that *eC* greatly impacts the physiology of C_3_ plants. However, the impacts of *eC* on the growth and physiology of C_4_ plants are less understood (Bowes, 1993). Until recently, it was supposed that C_4_ photosynthesis would not be affected by *eC* conditions, because intercellular [CO_2_] (*C_i_*) is saturated or near saturated values under *aC*. And there was weak evidence to support the opposite. However, several recent studies have demonstrated that photosynthesis rates of C_4_ plants are also increased in high [CO_2_] (Ainsworth and Long, 2021). Here, we demonstrated that enhanced photosynthesis under *eC* was associated with improved PSII performance (higher Y(II), qP, qL, and ETR) (**Figure 2A**; **Table 1**), suggesting that the extra carbon fixed under *eC* increased the efficiency of photosystems, corroborating the findings of Habermann et al. (2019b). Y(II) is the proportion of absorbed light that is being used in PSII photochemistry and used to calculate the electron transport rate through the PSII, whilst qL and qP estimate the fraction of open PSII centres (Walz, 2019). In this study, qP and qL increased due to the isolated effect of *eC*, while Y(II) and ETR increased due to additive effects of *eC* and *eT*, leading to an enhanced PSII performance under *eCeT* treatment (**Table 1**). Therefore, our results suggest that the PSII activity of *M. maximus* can acclimate to a warmer, CO_2_-enriched environment.

This improved photosynthetic performance under *eC* was accompanied by reduced *g_s_* and *E* values (**Figure 2B**). Smaller *g_s_* and *E* are widely reported to occur in other FACE studies, with no evidence indicating that this *g_s_* decrease acclimates with time (Ainsworth and Rogers, 2007; Ainsworth and Long, 2021). Smaller *g_s_* may occur due to short-term mechanisms such as stomatal closure (Ainsworth and Rogers, 2007) or long-term changes, such as decreases in stomatal density (Lin et al., 2001). The regulatory role of [CO_2_] in the differentiation process of the epidermic cells is well-recognized in the literature (Gray et al., 2000; Doheny-Adams et al., 2012; Engineer et al., 2014; Xu et al., 2016). For example, in a different study from this same experiment, Habermann et al. (2019b) showed that plants that developed under *eC* regardless of the temperature level had reduced stomatal density and stomatal index in both leaf surfaces.

One of the consequences of smaller *g_s_* and *E* is the modification of energy flux between canopy and environment. In this study, T_canopy_ of plants under *eCaT* was slightly higher when compared to *aCaT* treatment (**Figure 1C**), especially during the hottest hours of the day (between 11 am and 4 pm). This CO_2_-induced increase in crop temperature is a well-documented response associated with the smaller *g_s_* and *E* under *eC*, which decrease the cooling capacity of the canopy (Negi et al., 2014; Šigut et al., 2015). This [CO_2_] effect on T_canopy_ can approximate leaf temperature to the optimum temperature of photosynthesis or even exceed it, leading to some deleterious impacts on plant photosynthesis (Warren et al., 2011). However, in this study, there was no additive effect of [CO_2_] on T_canopy_ of plants grown under *eCeT* (**Figure 1C**). Interestingly, this additive effect has been found by our research team repeated times in different experiments conducted with the forage legume *Stylosanthes capitata* (Fabaceae, C_3_) under the combination of *eCeT* (Habermann et al., 2019a) and under *eT* combined with reduced soil moisture for *S. capitata* (Habermann et al., 2021) and *M. maximus* (Habermann et al., 2019c), but not here. To our knowledge this has not been described in the literature and would be crucial information to understand water use, heat stress and to improve management and the selection of future germplasm adapted different climate conditions. We speculate that this response can be associated with a higher evaporative demand under *eT* plots, cancelling the smaller *E* under *eC* (**Figure 1B**). This can also be associated with plant genotype being highly adapted to heat conditions and thus being able to function at a warmer T_canopy_ downregulating *g_s_*.

This differential stomatal response to *eC* and *eT* promoted significant impacts on the plant water status of *M. maximus*. Separating the direct effects of elevated temperature from some level of water stress induced by higher vapor pressure deficit (VPD) of the atmosphere is highly daunting (Xu et al., 2021). As expected, *eC* improved plant water status (**Figures 3**, **4**) presumably due to the reduced *g_s_* and *E* (Wullschleger et al., 2002), resulting in a smaller transpiration flux and water absorption from the soil (Nelson et al., 2004). Therefore, soil moisture under *eCaT* plots remained higher during the experimental period (**Figure 1A**). It has been reported that the conservation of soil moisture under *eC* can ameliorate the negative impacts of moderate water shortage periods (Nelson et al., 2004). However, in this study, this water conservation effect was completely offset by *eT* under *eCeT* treatment (**Figure 1A**). *eT* also promoted an antagonistic effect in plant water status presumably due to the intensification of leaf transpiratory rates. Therefore, our data suggest that a slight increase in temperature can mitigate the beneficial effect of *eC* on soil moisture conservation and plant water status.

The antagonistic effect between *eT* and *eC* was also observed for leaf proline content (**Figures 3E, F**, **4C, D**). The accumulation of protective compounds such as proline is often found to increase under heat stress (Haudecoeur et al., 2009; Szabados and Savoure, 2010). Here, *eT* increased leaf proline content, while *eC* decreased it, leading to contrary effects under combined conditions. Proline can act as reactive oxygen species (ROS) scavenger, protecting the structure of membranes and proteins, being essential in osmotic potential adjustment (Verbruggen and Hermans, 2008). In this study, we observed that MDA and chlorophyll contents were not modified by any treatment, and antioxidant enzymatic activity was mostly unaffected, except by a decrease in APX activity under *eT* (**Table 1**), suggesting a smaller investment in this antioxidant pathway defense system. Thus, the conditions of [CO_2_] and temperature tested in this study did not impose stressful conditions on plants.

Besides the impacts of *eC* and *eT* on the physiology and biochemistry of *M. maximus*, we investigated the impacts of both environmental factors on plant traits that are related to animal feeding (**Figures 5**, **6**). In pastures, aboveground productivity is one of the most important traits that determine milk and meat production. According to the study conducted by Carvalho et al. (2020) in the same experiment, *eC* alone did not increase belowground or aboveground biomass of *M. maximus* (**Supplementary Figure 2**), suggesting that surplus carbon assimilated under *eC* was partitioned to other structures or physiological processes, such as synthesis of secondary metabolites, respiratory metabolism, or the production of fibers. Interestingly, *eT* was the main responsible for increasing aboveground productivity at the final harvest (Carvalho et al., 2020) (**Supplementary Figure 2**). This higher biomass accumulation under *eT* was presumably related to higher photosystem efficiency (**Table 1**), greater root growth (Carvalho et al., 2020), sugar partitioning (Habermann et al., 2019c), and salicylic acid (SA) biosynthesis (Wedow et al., 2019). In addition, higher biomass productivity under *eT* is expect to increase the demand for more N, Ca, and S of *M. maximus* plants (Carvalho et al., 2020). However, this *eT*-induced improvement in aboveground biomass is only observed when soil moisture is not a limiting factor (Viciedo et al., 2019).

Another important response observed in our study is the decrease of aboveground nutritive value and digestibility of *M. maximus* pasture (**Figures 5**, **6**). Here, the aboveground fibrous fraction (NDF content) increased by *eC* and *eT* effects, leading to an additive increase under *eCeT*, whilst ADF increased due to isolated *eT* effect. ADF and NDF are anti-quality components of forages that reduces the digestibility of plant material and the amount of energy that animals obtain from forages (Waghorn and Clark, 2004). Moreover, forages with high fiber content increase the satiety level of animals, reducing animal feeding (Allen, 1996; Dias et al., 2021). The additive pattern of response promoted by *eC* and *eT* was also observed for lignin synthesis. Our results indicate that part of the additional carbon assimilated under *eC* was presumably being partitioned to the production of fibers. This is the first study that demonstrated that higher levels of [CO_2_] also contribute to reducing forage quality and digestibility of *M. maximus* and that *eC* and *eT* synergize to amplify this negative effect on forage traits.

Lignification process is highly dependent on temperature (Nahar et al., 2015). For instance, forages from warmer regions such as tropical zones are poorer in quality when compared to forages from temperate zones, since elevated temperature is reported as an important factor in increasing the lignification level of plant tissues (Ball et al., 2001; Lee, 2018). Lignin limits the digestion of plant material because it represents a physical barrier against the digestive enzymes from microorganisms in the rumen (Dumont et al., 2015). This *eT*-induced decrease of *M. maximus* forage quality was previously reported by Habermann et al. (2019c); Habermann et al. (2022) under well-watered and rainfed conditions. Evidence indicates that the increase of leaf fibrous fraction and lignin contents of *M. maximus* are not correlated with the proportion of any leaf tissue, suggesting that leaf chemical composition, not leaf tissue proportion, was the main factor reducing forage quality (Habermann et al., 2022). This hypothesis was also corroborated by Wedow et al. (2019), where authors showed that *M. maximus* plants developed under elevated temperature (2°C) had a higher concentration of synaptic acid and tocopherol, two intermediates of the lignin synthesis pathway, suggesting that lignin composition is being modified by *eT*.

Forages rich in proteins and minerals are essential to sustain cattle performance, health, and productivity (Moyo and Nsahlai, 2021). In this study, we observed an additive decrease of CP due to *eC* and *eT*, dropping protein levels from approximately 20% under *aCaT* to 15% under *eCeT*. Protein is one of the most important nutrients that animals obtain from plant material. Forages with low protein content cannot sustain the metabolism of microorganisms responsible for the digestion of plant material, resulting in a smaller ruminant’s intake and forage digestibility (Bach et al., 2005). Crude protein content also declines with rising temperatures and fiber content, as observed here (**Figures 5**, **6**). This reduced quality of forages under future conditions of temperature and CO_2_ is a matter of concern since other abiotic conditions such as increased tropospheric ozone concentration (Hayes et al., 2016) and reduced soil moisture (Habermann et al., 2019c; Habermann et al., 2021) also can promote a reduction in forage quality, suggesting that when all those factors are combined more intense impacts on forages may be expected. However, there is a need for multifactorial studies that combine all those factors to test this hypothesis.

## Conclusion

Our results, based on one grazing growth cycle, showed that high atmospheric [CO_2_] and warming have antagonistic effects on *M. maximus* plant water relations. In summary, we observed that plants fixed more carbon using fewer water resources under *eC* conditions, conserving more water in the soil and plant tissues. However, this water conservation effect was offset by warming. Plant metabolism was not harmed by *eC* or *eT* conditions, instead, PSII photochemistry improved due to the additive effects of both factors. Both high atmospheric [CO_2_] and warming synergized to increase fibrous fraction and lignin and decrease the digestibility and crude protein of this common tropical forage grass, with potential consequences for animal feeding and the global carbon cycle like an amplification in methane emissions by ruminants, driving positive carbon-climate feedback (Knapp et al., 2014; Wollenberg et al., 2016). We highlight the need for more multifactorial and long-term field studies to better elucidate how tropical grasses will be affected by future global change conditions.

## Data availability statement

The original contributions presented in the study are included in the article/**Supplementary Material**. Further inquiries can be directed to the corresponding author.

## Author contributions

EH and ED wrote the manuscript. EH, CM, ED, and JC collected data in the field and processed the data. CM and KP conceived and designed the experiments. All authors contributed to the revision of the manuscript. All authors contributed to the article and approved the submitted version.

## Funding

This work was supported by the Sao Paulo Research Foundation, FAPESP Thematic Project (Grant 08/58075-8) to CM. FAPESP provided graduate studentships to EH (Grant 14/26821-3; 16/09742-8) and ED (Grant 14/00317-7). CNPq provided a scholarship to DC (Grant 385485/2015-8). CM received financial support from CNPq (Process 446357/2015-4). CM is a research fellow from CNPq (Grant 302628/2019-3).

## Acknowledgments

We thank Bruce Kimball from the USDA and Franco Miglietta from IBIMET, Italy. The authors thank Wolf Seeds from Ribeirão Preto, São Paulo State, Brazil, for providing seeds of *M. maximus*. We thank graduated and undergraduate students that contributed in the field work. We thank the Goiano Federal Institute for the support provided for bromatological analysis.

## Conflict of interest

The authors declare that the research was conducted in the absence of any commercial or financial relationships that could be construed as a potential conflict of interest.

The reviewer AG declared a shared affiliation with the authors EH, ED, DC, CM to the handling editor at the time of the review.

## Publisher’s note

All claims expressed in this article are solely those of the authors and do not necessarily represent those of their affiliated organizations, or those of the publisher, the editors and the reviewers. Any product that may be evaluated in this article, or claim that may be made by its manufacturer, is not guaranteed or endorsed by the publisher.
