# Supplementary material for: Warming offsets the benefits of elevated CO2 in water relations while amplifies elevated CO2-induced reduction in forage nutritional value in the C4 grass Megathyrsus maximus
Source: Front Plant Sci. 2022 Dec 5;13:1033953. doi: 10.3389/fpls.2022.1033953 (PMC9760913; doi:10.3389/fpls.2022.1033953)
Supplement: Supplementary file 1 [file DataSheet_1.docx]

Supplementary Material

**Supplementary figure 1**


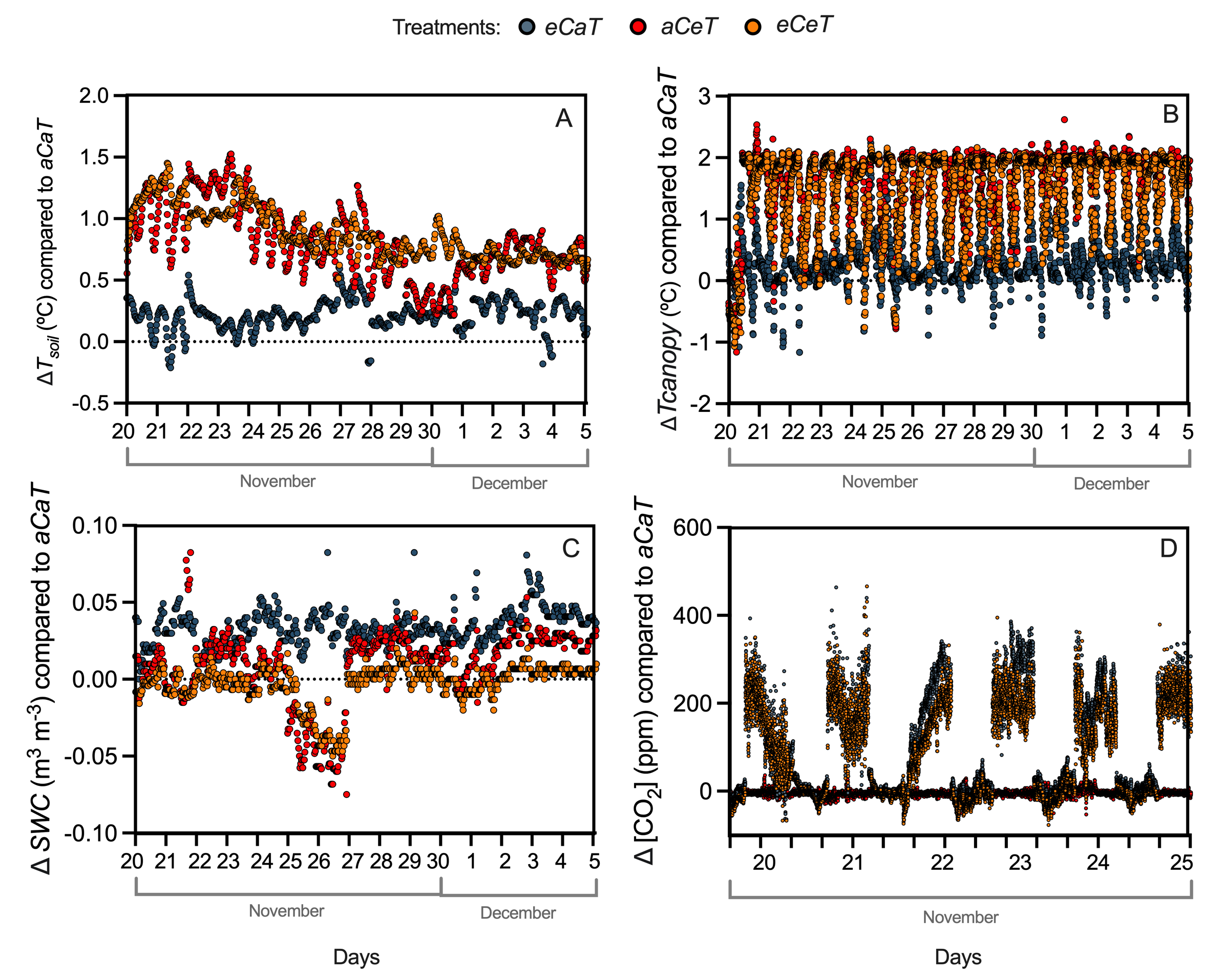


**Supplementary Figure 1**. Difference (Δ) in microclimate conditions registered in each treatment compared to ambient conditions (*aCaT*) during the experimental period conducted with *M. maximus* under different levels of [CO_2_] and temperature. (A) *T_soil_* = soil temperature. (B) *Tcanopy* = canopy temperature. (C) *SWC* = Soil water content. (D) [CO_2_] = atmospheric CO_2_ concentration. Points were obtained each hour for *SWC* and *Tsoil*, each 15 minutes for *Tcanopy* along the entire experiment and [CO_2_] each minute along six days of the experimental period. Treatments: *aC* (ambient CO_2_ concentration), *eC* (elevated CO_2_ concentration – 600 ppm), *aT* (ambient temperature), and *eT* (elevated temperature - 2°C above ambient temperature).
