# Supplementary material for: Warming offsets the benefits of elevated CO2 in water relations while amplifies elevated CO2-induced reduction in forage nutritional value in the C4 grass Megathyrsus maximus
Source: Front Plant Sci. 2022 Dec 5;13:1033953. doi: 10.3389/fpls.2022.1033953 (PMC9760913; doi:10.3389/fpls.2022.1033953)
Supplement: Supplementary file 2 [file DataSheet_2.docx]

Supplementary Material

**Supplementary figure 2**


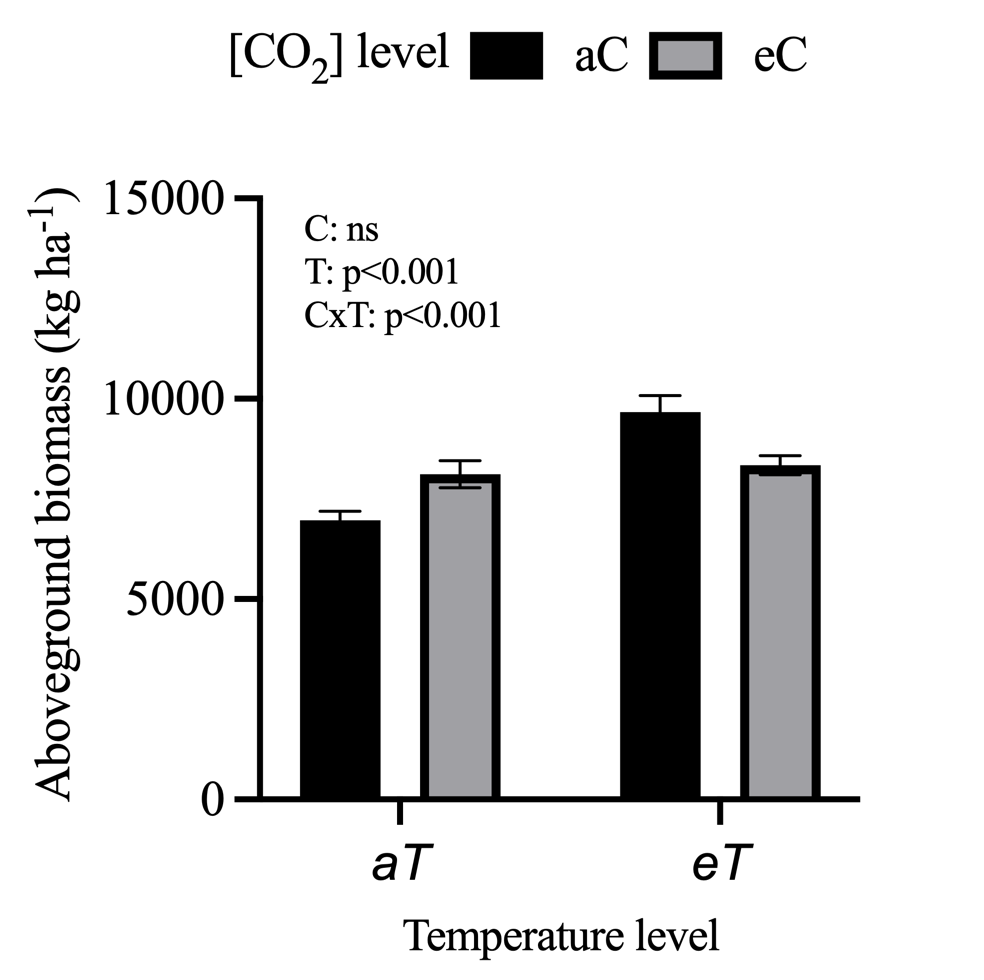


**Supplementary Figure 2**. Aboveground biomass production in the end of the experimental period (30 days after treatments started) conducted with *M. maximus* under different levels of [CO_2_] and temperature. Data were extracted from Carvalho et al. (2020), where a more detailed analysis of biomass production along the experiment can be found. Treatments: *aC* (ambient CO_2_ concentration), *eC* (elevated CO_2_ concentration – 600 ppm), *aT* (ambient temperature), and *eT* (elevated temperature - 2°C above ambient temperature). ANOVA results are shown for diurnal averages: C: [CO_2_] effect, T: temperature effect, C×T: interaction between C and T. ns = non-significant.
