# Supplementary material for: Warming offsets the benefits of elevated CO2 in water relations while amplifies elevated CO2-induced reduction in forage nutritional value in the C4 grass Megathyrsus maximus
Source: Front Plant Sci. 2022 Dec 5;13:1033953. doi: 10.3389/fpls.2022.1033953 (PMC9760913; doi:10.3389/fpls.2022.1033953)

Supplementary Material

**Supplementary Table 1**. Additional statistical analysis of leaf gas exchange parameters measured in fully expanded leaves of *M. maximus* in different levels of [CO_2_] and temperature. A = net photosynthesis rate (μmol m^-2^ s^-1^). g_s_ = stomatal conductance (mol m^-2^ s^-1^). E = transpiration rate (mmol m^-2^ s^-1^). Treatments: *aC* (ambient CO_2_ concentration), *eC* (elevated CO_2_ concentration – 600 ppm), *aT* (ambient temperature), and *eT* (elevated temperature - 2°C above ambient temperature). Two-way ANOVA results are shown for each parameter. F values and P values are shown. * = P<0.05, ** = P<0.01, *** = P<0.001, ns = non-significant. F_C_: [CO_2_] effect, F_T_: temperature effect, F_C:T_: interaction between [CO_2_] and temperature.


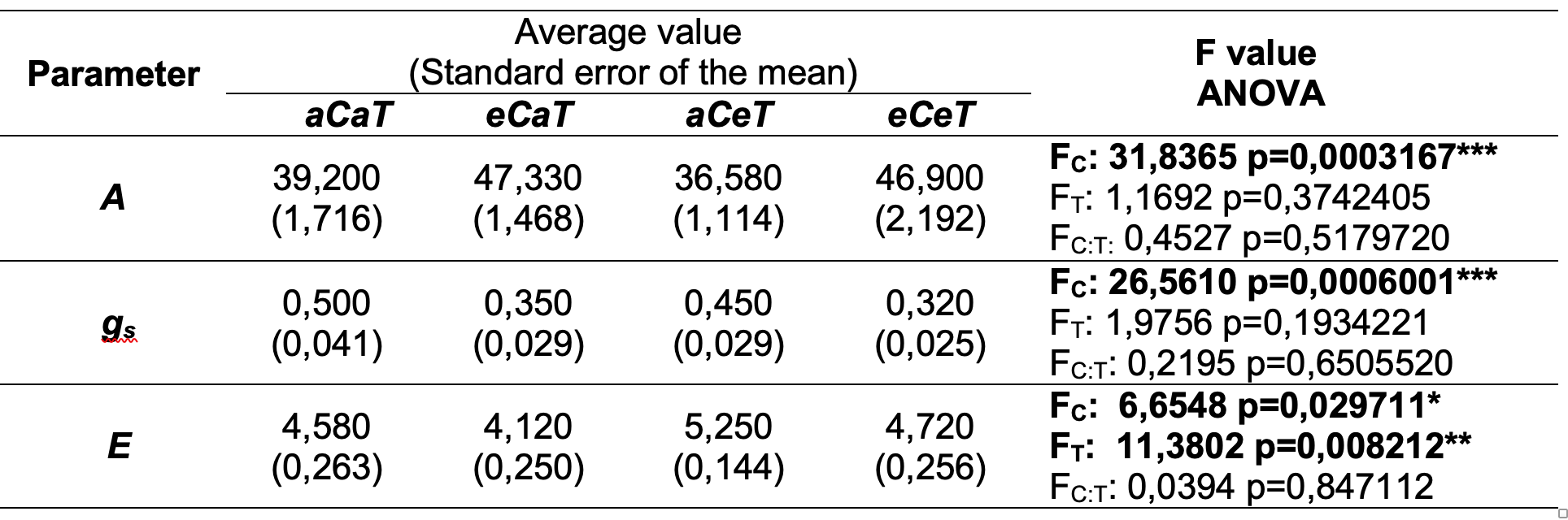

Supplement: Supplementary file 3 [file Table_1.docx]
